# White matter microstructure associations with episodic memory in adults with Down syndrome: a tract-based spatial statistics study

**DOI:** 10.1186/s11689-021-09366-1

**Published:** 2021-04-20

**Authors:** Austin Bazydlo, Matthew Zammit, Minjie Wu, Douglas Dean, Sterling Johnson, Dana Tudorascu, Ann Cohen, Karly Cody, Beau Ances, Charles Laymon, William Klunk, Shahid Zaman, Benjamin Handen, Andrew Alexander, Bradley Christian, Sigan Hartley

**Affiliations:** 1 School of Medicine and Public Health, University of Wisconsin-Madison, Madison, WI USA; 2 University of Pittsburgh School of Medicine, Pittsburgh, PA USA; 3 Wisconsin Alzheimer’s Disease Research Center, University of Wisconsin-Madison, Madison, WI USA; 4 Waisman Center, University of Wisconsin-Madison, Madison, WI USA; 5 Washington University of St. Louis, St. Louis, MO USA; 6 Cambridge Intellectual and Developmental Disabilities Research Group, University of Cambridge, Cambridge, UK; 7 School of Human Ecology, University of Wisconsin-Madison, Madison, WI USA

## Abstract

**Background:**

Nearly all persons with Down syndrome will show pathology of Alzheimer’s disease in their 40s. There is a critical need for studies to identify early biomarkers of these various pathological changes of Alzheimer’s disease in the Down syndrome population and understand the relationship of these biomarkers to cognitive symptoms in order to inform clinical trials. Although Alzheimer’s disease is often considered a disease of gray matter, white matter degeneration has been documented during the preclinical stage of Alzheimer’s disease. The current study examined the association between diffusion tensor imaging (DTI) measures of white matter microstructure and episodic memory performance in 52 adults with Down syndrome.

**Methods:**

Seventy (*N* = 70) participants (M = 40.13, SD = 7.77 years) received baseline scans as part of the Neurodegeneration in Aging Down Syndrome (NiAD) study at two imaging facilities (36 at the University of Wisconsin-Madison [UW-Madison] and 34 at the University of Pittsburgh Medical Center [UPMC]). All participants had genetically confirmed trisomy 21. Fifty-two (*N* = 52) participants remained after QC. The DTI measures, fractional anisotropy (FA) and mean diffusivity (MD), were calculated for each participant. A combined measure of episodic memory was generated by summing the z-scores of (1) Free and Cued Recall test and (2) Rivermead Behavioural Memory Test for Children Picture Recognition. The DTI data were projected onto a population-derived FA skeleton and tract-based spatial statistics analysis was conducted using the FSL tool PALM to calculate Pearson’s *r* values between FA and MD with episodic memory.

**Results:**

A positive correlation of episodic memory with FA and a negative correlation of episodic memory and MD in the major association white matter tracts were observed. Results were significant (*p* < 0.05) after correction for chronological age, imaging site, and premorbid cognitive ability.

**Conclusion:**

These findings suggest that white matter degeneration may be implicated in early episodic memory declines prior to the onset of dementia in adults with Down syndrome. Further, our findings suggest a coupling of episodic memory and white matter microstructure independent of chronological age.

**Supplementary Information:**

The online version contains supplementary material available at 10.1186/s11689-021-09366-1.

## Background

Down syndrome (DS) is a genetic disorder caused by full or partial trisomy 21 and is associated with a host of physical and developmental problems including intellectual disability, congenital heart disease, hypotonia, thyroid disorders, and sleep problems [1]. People with DS also have several neuroanatomical brain differences from typically developing populations, including brachycephaly, ventriculomegaly, regional hypoplasia, and decreased depth and reduced number of cerebral sulci [2]. DS is also associated with an increased prevalence and early onset of Alzheimer’s disease (AD) purportedly due to the triplication of the amyloid precursor protein (APP) gene, located on chromosome 21, which causes an over-expression of amyloid-beta (Aβ). The extracellular accumulation of Aβ plaques in the brain is one of the early hallmarks of AD [3, 4]. The genetic linkage of increased amyloid production in DS to increased AD risk is most similar to the development of autosomal dominant AD in non-DS populations, which account for only 1% of AD cases as opposed to sporadic AD which accounts for 90% of total cases [5, 6]. Population-based studies suggest that over half of adults with DS aged 55 years and older receive a clinical AD diagnosis [7], with the lifetime risk of clinical AD in DS is approximately 90% [8]. There is a critical need for natural history studies to characterize early biomarkers of pathological changes of Alzheimer’s disease in the Down syndrome population and understand the relationship of these biomarkers to cognitive declines in order to inform clinical trials aimed at delaying or preventing AD in this at-risk population [9].

Positron emission tomography (PET) imaging studies have recently been used to characterize the early accumulation of Aβ using the biomarker [^11^C] Pittsburgh Compound-B (PiB) in adults with DS prior to onset of clinical AD. These studies found that a subset of adults with DS evidence marked Aβ accumulation (referred to as PiB (+)), typically beginning in the striatum, by their fourth decade of life with the majority being PiB (+) by the middle of their fifth decade [10, 11]. These findings indicate PET imaged Aβ accumulation occurs approximately 30 years earlier in the DS population as compared to general population samples of adults without a genetic risk for AD [10, 11].

Although AD is often considered a disease of gray matter, white matter (WM) degeneration is evident in adults diagnosed with clinical AD [12–14] and is also observed during the preclinical stage (i.e., prior to onset of clinical AD) and thought to result from the presence of extracellular Aβ plaques followed by intracellular neurofibrillary tangles of the protein tau [13–16]. Diffusion tensor imaging (DTI) is a non-invasive magnetic resonance imaging (MRI) technique that probes microstructural differences in water diffusion properties of biological tissue [4, 17–19] and has been used to examine WM change associated with AD in both the DS [9, 20] and non-DS populations [21, 22]. DTI measurements include the mean diffusivity (MD), which is the directionally averaged diffusivity and is sensitive to the density of microstructural features; and the fractional anisotropy (FA), which is a summary measure of the directional variance of diffusivities and is often used as a sensitive marker of WM microstructural changes [23].

Across studies of non-DS populations, adults with clinical AD have been found to evidence increased MD and decreased FA across multiple brain regions [12, 13, 16]. Moreover, WM integrity has been found to be associated with cognitive decline and, more specifically, impaired episodic memory in non-demented adults from non-DS populations [12, 15, 24]. For example, Nicholas et al. (2020) found that increases in frontal MD across time were associated with decreases in the ability to correctly recall words (i.e., free recall) in older adults without AD [25]. Remy et al. (2015) found an association between lower FA in the medial temporal lobe and worse episodic memory in older adults without AD [26]. Similarly, Metzler-Baddeley et al. (2011) showed that better WM integrity (increased MD and decreased FA) in the fronto-temporal lobe was associated with better episodic memory in older adults without AD [27]. Lastly, Lockhart et al. (2012) found associations between lower FA and worse episodic memory across younger and older adults without AD throughout major association fibers, including the superior longitudinal fasciculus, inferior longitudinal fasciculus, the cingulum bundles, uncinate fasciculus, and thalamo-cortical projections [28].

In previous work from our own research group, we found evidence that WM integrity may also be impacted early on in AD in the DS population. Specifically, WM integrity assessed using DTI was negatively associated with PET Aβ using PiB in 65 non-demented adults with DS. Adults with DS who were PiB (+) in the striatum and/or neocortex had a higher level of WM insult than did those without marked accumulation (PiB (−)). These differences occurred for both MD and FA along the major association tracts including superior longitudinal fasciculus, inferior longitudinal fasciculus, the cingulum, and uncinate fasciculus [29, 30]. Building on these findings, it is now important to determine if WM integrity is associated with declines in episodic memory, which also occur early on in the transition to dementia in DS [24, 31, 32] and thus may be an informative biomarker of the transition to the prodromal stage of AD in DS.

Given the large number of neuroanatomical abnormalities in DS and the role of overproduction of Aβ in the development of AD in DS, it is not clear if WM impairments are involved early on in AD in DS and if DTI is a meaningful biomarker of WM differences linked to AD-related cognitive change in this population. The goal of the present study was to examine the association between DTI measured WM integrity and directly administered measures of episodic memory in 52 adults with DS. We hypothesized that WM integrity, as characterized by FA and MD in the major association tracts (superior longitudinal fasciculus, inferior longitudinal fasciculus, the cingulum, and uncinate fasciculus), would be associated with episodic memory performance. Analyses were conducted with and without controlling for chronological age and premorbid cognitive ability. Given that data come from two study sites, we also added a control variable for site in analyses.

## Methods

### Participants

Consent or assent for study participation was obtained from all adults with DS. Proxy consent was obtained from caregivers who served as legal guardians. This study was performed under the approval of the institutional review boards for human subjects research. Seventy (*N* = 70) participants (M = 40.13, SD = 7.77 years) received baseline scans as part of the Neurodegeneration in Aging Down Syndrome (NiAD) study at two imaging facilities (36 at the University of Wisconsin-Madison [UW-Madison] and 34 at the University of Pittsburgh Medical Center [UPMC]). All participants had genetically confirmed trisomy 21. Subject demographic information is presented in Table 1. Eighteen (*n* = 18) participants were excluded due to excessive motion during the DTI acquisition and removed from analyses. Of the remaining participants, forty-six (*n* = 46) participants were classified cognitively stable and three (*n* = 3) were classified as having mild cognitive impairment (MCI) and three (*n* = 3) were diagnosed with dementia. These clinical status determinations were based on a case consensus process that included at least three staff with clinical expertise who were blind to MRI and PET imaging data. The following information was used in the case consensus process: (a) medical/psychiatric history and neurological exam; (b) caregiver-report of participant’s functioning and life events; (c) participant’s adaptive skills on the Vineland Adaptive Behavior Scales [33]; (d) caregiver-report of participant’s dementia symptoms on Dementia Questionnaire for People with Learning Disabilities [34] or Dementia Scale for Down syndrome [35]; (e) participant’s profile on the Down Syndrome Mental Status Examination [36], Developmental Test of Visual-Motor Integration, 5th Edition [37], Wechsler Intelligence Scale for Children [38], Block Design and Haxby extension [35], and Developmental NEuroPSYchological Assessment [39] Word Generation Semantic Fluency. Informed consent was obtained prior to data collection.

**Table 1:** Subject demographic information

	Total (*N* = 52)	MCI (*N* = 3)	Dementia (*N* = 3)
Subjects imaged at UW	36	3	0
Subjects imaged at UPMC	34	0	3
Females (%)	24 (46.15%)	0 (0%)	1 (33%)
Age in years (SD)	39.13 (7.77)	45.53 (3.59)	50.47 (5.26)
RSPPV (SD)	122.20 (34.63)	131.33 (24.58)	84.00 (17.78)

### Diffusion tensor imaging

Magnetic resonance imaging (MRI) data were collected on two 3.0T MRI scanners—a GE SIGNA 750 with an 8-channel head coil (UW-Madison) and a Siemens Magnetom Trio scanner with a 64-channel head coil (UPMC). Diffusion-weighted imaging at both sites was performed using a single-shell, diffusion-weighted spin echo sequence (UW-Madison TR/TE = 7800/67 ms; UPMC TR/TE = 7200/56 ms). The DWI protocol consisted of either 7 (UPMC) or 6 (UW-Madison) non-diffusion weighted (*b*_0_) images and diffusion weighted images with a *b* value of 1000 s/mm^2^ in 48 non-collinear directions. Additional imaging parameters consisted of matrix size: and 116 × 116 with 80 slices, field of view: 23.2 × 23.2 × 16 cm^3^, and 2 mm slice thickness. Data were processed using an in-house processing pipeline utilizing tools from FSL [40], Mrtrix [41], and the DiPy toolbox [42]. The diffusion-weighted data were corrected for Gibbs’ ringing artifacts [43], Rician noise [44], and eddy current distortions and head motion with outlier replacement [45, 46]. A threshold of 10% or more of slices replaced as outliers within a single diffusion weighted image (DWI) was established as a criterion for removal of a volume; however, no volumes exceeded this threshold and no DWIs were removed. The diffusion tensors were estimated using a robust estimator method, RESTORE [47], and FA and MD maps subsequently calculated.

All FA data in the study were aligned to a 2 mm isotropic population-derived FA template using ANTs [48]. This template was constructed using amyloid negative participants without MCI or AD. A medial surface skeleton was generated from the population-averaged FA image. Each participant’s regional maximum FA data were then projected onto this skeleton surface for voxel-wise statistical analyses. The MD maps were likewise spatially normalized and projected onto the FA skeleton for analysis. The JHU white matter atlas labels [49] were warped to the population-derived FA template using ANTs [48].

### Episodic memory composite measure

A composite score of episodic memory was calculated based on two measures of episodic memory. The first was the Free and Cued Recall test [50], a measure of verbal episodic memory in which participants attempt to learn and remember 12 pictures that are linked to categories (e.g., fruit). The Free and Cued Recall score is the number of pictures recalled across three free trials and three cued recall trials (i.e., category is given). The second measure was the Rivermead Behavioural Memory Test for Children Picture Recognition [51], a measure of visual episodic memory obtained by determining if participants are able to distinguish 10 pictures previously presented from 10 pictures not previously seen after a brief delay. The total score is the number correctly recalled minus the number of false positives. These two scores (Free and Cued Recall total and Rivermead Total) were *z*-scored and summed to create the composite measure used in the present study. The decision to use this composite measure of episodic memory was based on a principal component analysis involving five measures—Free and Cued Recall, Rivermead, Developmental NEuroPSYchological Assessment (NEPSY) Visual Attention and Verbal Fluency tasks [39], and the Beery Visual-Motor Integration [37]. The first principal component was made up of the Free and Cued Recall, and Rivermead scores and explained over 50% of the variance. Moreover, both measures have been shown to be sensitive to other biomarkers of early AD in adults with DS (e.g., Hartley et al. 2014).

### Control variables

Sociodemographic variables and study site were included in models to account for any effect on WM and episodic memory. Chronological age in years was reported by caregivers and is a useful control measure due to the coupling of DTI measures with age [52]. Premorbid (i.e., prior to any concerns of AD) cognitive ability was assessed using the Peabody Picture Vocabulary Test [53], which assesses receptive language ability and has been shown to be strongly associated with lifetime global cognitive ability [54]. Imaging site was coded as Wisconsin = 1 and Pittsburgh = 2 to allow us to control for any differences in WM or episodic memory based on site. The use of a site covariate was added to control for scanner-related differences in the imaging protocol.

### Statistical analyses

The distribution of variables and histograms of residuals were used to assess use the normality of data and to identify any outliers. Statistical analyses of the DTI data were performed using the tract-based spatial statistics pipeline in FSL [55, 56].

Pearson partial correlation analyses were performed using a general linear model in FSL. The model consisted of the episodic memory composite score, as well as the following control variables—chronological age, imaging site, and premorbid cognitive ability. Voxel-wise partial correlation analysis of the tract-based spatial statistics (TBSS)-derived data and episodic memory composite score (EMCS) using the above GLM was performed using the permutation analysis of linear models (PALM) package from FSL with 2D threshold-free cluster enhancement (TFCE) optimization [57]. Correction for multiple comparisons was performed by controlling for the family-wise error rate (FWER) [58].

## Results

**Fig. 1:**
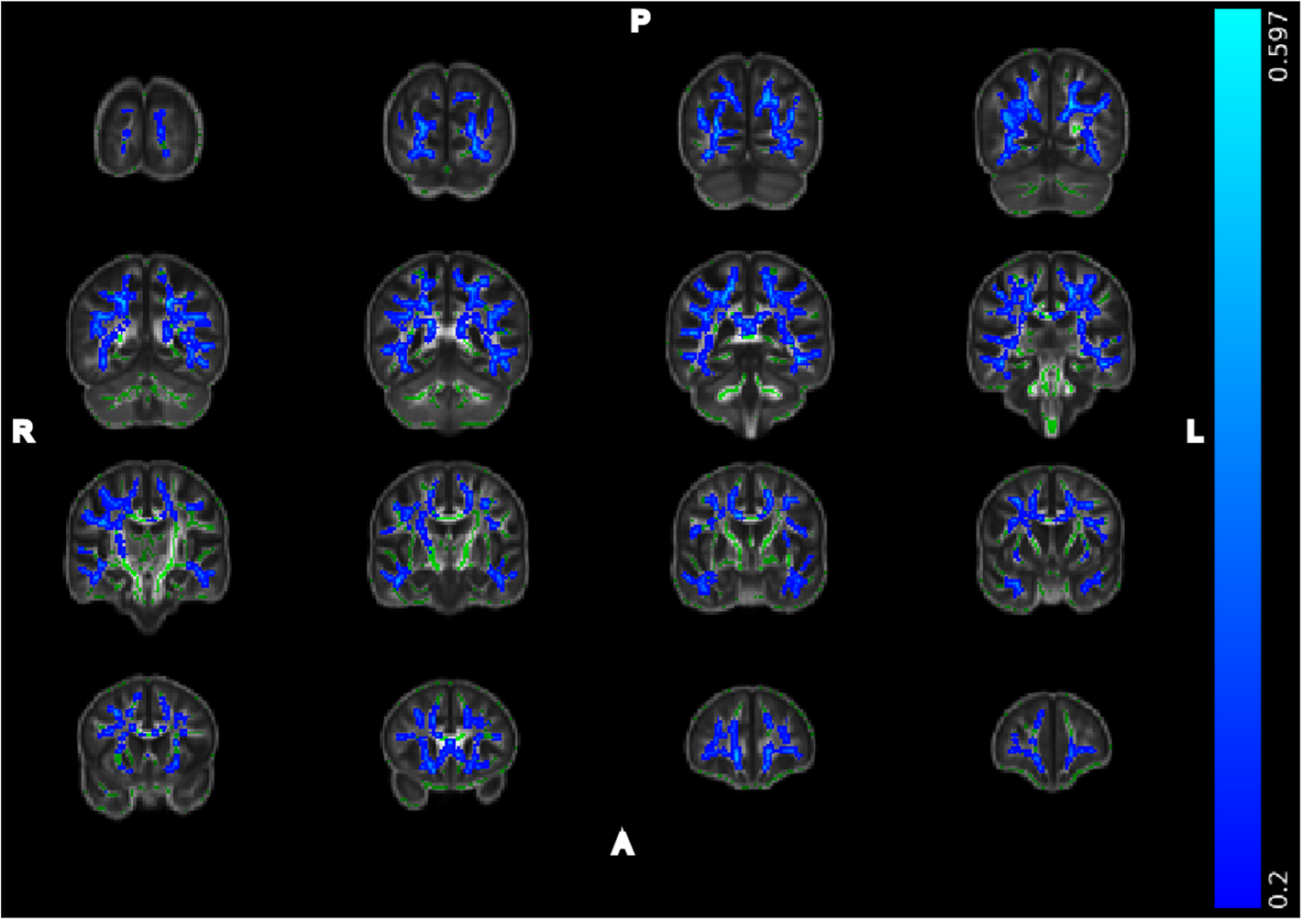
Regions of significant negative correlation between EMCS and MD. Images arranged in right to left (R-L) and posterior to anterior (P-A). Images overlaid on the population-derived FA skeleton (green) and the population-derived 2 mm FA template. Regions shown reflect areas with Pearson’s *r* less than *−* 0.2. Data shown at a FWER corrected *p* < 0.05 with covariates for premorbid cognitive ability, imaging site, and age

The episodic memory score was found to be near normal (kurtosis = 0.460) and with minimal skew (Pearson’s mode skewness = − 1.270). Figure 1 shows the significant negative correlations between the episodic memory composite with MD. MD data are shown at a multiple comparisons corrected significance of *p* < 0.05, with additional covariates for imaging site, premorbid cognitive ability, and chronological age. These covariates were used to address variation between scanners (imaging site), the understanding of the task (premorbid cognitive ability), and the association of chronological age and DTI measures. Significant associations were observed bilaterally throughout the superior and inferior association fibers (see Table 2): superior longitudinal fasciculus (left: *r* = − 0.399, *p* = 0.005 FWER corrected; right: *r* = − 0.444, *p* = 0.001 FWER corrected) as well as in the inferior longitudinal fasciculus (left: *r* = − 0.504, *p* < 0.001 FWER corrected; right: *r* = − 0.452, *p* = 0.001 FWER corrected). We also examined FA within these regions and found significant associations bilaterally in the superior longitudinal fasciculus (left: *r* = 0.280, *p* = 0.051 FWER corrected; right: *r* = 0.332, *p* = 0.020 FWER corrected) as well as in the inferior longitudinal fasciculus (left: *r* = 0.292, *p* = 0.042 FWER corrected; right: *r* = 0.372, *p* = 0.008 FWER corrected). All reported ROIs were extracted using a mask of the significant voxels reported in Fig. 1 within JHU WM atlas labels provided by FSL [40, 49]. No significant negative correlations with FA or positive associations with MD were observed. Note that data are presented using the “tbss_fill” method provided by FSL, and inflated regions reflect areas significant at a multiple comparisons corrected *p* < 0.05. As a follow-up analysis, we re-ran the above model after removing the subjects with MCI or AD. The overall pattern of results remained the same. The inferior longitudinal fasciculus MD (left: *r* = − 0.451 (*p* = 0.002); right: *r* = − 0.339 (*p* = 0.026)), superior longitudinal fasciculus MD (right: *r* = − .305 (*p* = 0.047)), inferior longitudinal fasciculus FA (left: *r* = 0.292 (*p* = 0.057); right: *r* = 0.407 (0.007)), and superior longitudinal fasciculus FA (left: *r* = 0.28 (*p* = 0.076); right: *r* = 0.370 (*p* = 0.015)) regions remained significantly associated with episodic memory after correction for chronological age, site, and premorbid cognitive ability. The global regions (FA: *r* = 0.201 (*p* = 0.196); MD: *r* = − 0.376 (*p* = 0.013)) had trend-level associations with episodic memory after correcting for chronological age, site, and premorbid cognitive ability. Thus, the inclusion of the MCI and AD participants did not appear to be driving findings.

**Table 2:** JHU atlas ROI comparisons of DTI parameters and the episodic memory composite score (EMCS)

	Pearson’s *r* (*p* value*)	
Global FA and EMCS	0.341 (0.017)	
Global MD and EMCS	− 0.547 (< 0.001)	
	Left hemisphere	Right hemisphere
ILF FA and EMCS	0.292 (0.042)	0.372 (0.008)
SLF FA and EMCS	0.280 (0.051)	0.332 (0.020)
ILF MD and EMCS	− 0.504 (< 0.001)	− 0.452 (0.001)
SLF MD and EMCS	− 0.399 (0.005)	− 0.444 (0.001)

*Corrected for family-wise error rate, imaging site, chronological age, and premorbid cognitive ability

Though voxelwise associations with FA did not survived at an α = 0.05 level, positive correlations between FA and EMCS were observed at *p* < 0.08 corrected for chronological age, imaging site, and premorbid cognitive ability (see Supplementary Figure 1A). We also tested the correlation between the composite measure and FA only covarying for imaging site and premorbid cognitive ability and observed diffuse regions of positive correlation of the composite score with FA at *p* < 0.05 FWER corrected (see Supplementary Figure 1B). The extent of these regions throughout the association tracts is quite similar to the extent observed for MD in Fig. 1.

## Discussion

Adults with DS are genetically at-risk for AD with AD-related pathophysiology nearly universally present by age 40 years [11]. There is a critical need within the DS field for imaging studies that can describe the natural history of early AD pathophysiology in DS and its link to cognitive decline in order to inform clinical trials. To our knowledge, the present study is the first to examine the association between DTI measured WM integrity and episodic memory in adults with DS.

Our findings revealed important associations between WM integrity and episodic memory in the DS population. FA was observed to be positively correlated with episodic memory while MD was negatively correlated with episodic memory. There were no significant negative associations between FA and episodic memory nor positive associations between MD and episodic memory. Associations between FA and MD (in positive and negative directions respectively) and worse performance on measures of cognitive ability including episodic memory have been reported on extensively in the aging literature on non-DS populations [26–28]. Further, decreased FA and/or increased MD have been reported in adults without DS who are exhibiting MCI and/or AD relative to healthy controls [20, 59]. Our findings serve as a bridge between the cognitive insights of bridge between the previous reports of the central role of episodic memory declines in AD in DS [60–62] with the body of DS DTI literature [9, 20, 59] showing that disruptions of episodic memory in early cognitive decline may arise from the degeneration of association white matter pathways between regions of the brain, particularly frontal, medial-temporal, and parietal lobe areas. Further, our results highlight the clinical potential of DTI, particularly MD, as a non-invasive biomarker to detect early cytoarchitectural changes that may be associated with AD in adults with DS. If DTI continues to emerge as a useful biomarker of AD-related cognitive change in future studies that are larger and longitudinal, this biomarker has the potential to be informative for participant selection and outcome tracking in AD clinical trials in DS. Indeed, efforts to launch large AD intervention trials in DS are already underway, yet currently involve a limited array of established AD biomarkers. DTI is already an established biomarker in pediatric populations and is used to detect both gray matter and white matter changes arising from neurodevelopmental conditions such as Gaucher’s disease [63] and Crigler-Najjar Syndrome [64], illustrating the promise of DTI as a clinically relevant biomarker in rare genetic disorders, such as DS.

There is a tight linkage between chronological age and DTI indices which may have impacted our results [52, 65]. Indeed, in the current sample, chronological age was correlated with FA in major tracts at *r* = − 0.482 (*p* < 0.001). However, the FA and MD associations with episodic memory remained even after controlling for chronological age. There is also a coupling of episodic memory performance with premorbid cognitive ability (*r* = 0.508, *p* < 0.001) and decline with chronological age (*r* = − 0.491, *p* < 0.001) in our sample. In controlling for chronological age and premorbid cognitive ability, our findings represent the episodic memory changes above and beyond these other effects. Our results suggest a coupling of white matter cytoarchitecture and episodic memory that is independent of chronological age. Further study is needed to understand other factors that may impact this relation.

## Conclusion

The present study had both strengths and limitations. To our knowledge, this is the largest study examining the association between DTI measures of WM integrity and cognitive functioning in the DS population and is the first to show that WM impairment may be implicated in early declines in episodic memory in DS. There were also limitations to the present study. First, adults with DS are a difficult population to image, due to increased subject motion in the scanner. Indeed, motion led to the rejection of several subjects due to imaging artifacts. Second, larger cohorts and longitudinal studies are needed to tease apart the time-ordered direction of effects of WM impairment and episodic memory with age, as this is not clear given the cross-sectional nature of the present study. Finally, future studies should include biomarkers of Aβ and tau in addition to WM integrity and evaluate whether WM impairment has an effect on cognitive functioning that is independent from these other aspects of AD pathophysiology.

## Supplementary Information

**Additional file 1: Supplementary Figure 1.** A) Regions of significant positive correlation between EMCS and FA at *p* < 0.08 FWER corrected and controlling for chronological age, imaging site, and premorbid cognitive ability. B) Regions of significant positive correlation between EMCS and FA at *p* < 0.05 FWER corrected and controlling for imaging site and premorbid cognitive ability. Images arranged in right to left (R-L) and posterior to anterior (P-A). Images overlaid on the population derived FA skeleton (green) and the population derived 2mm FA template.

## Data Availability

The datasets used and/or analyzed during the current study are available from the corresponding author on reasonable request.
