# Supplementary figures and images for: White matter microstructure associations with episodic memory in adults with Down syndrome: a tract-based spatial statistics study
Source: J Neurodev Disord. 2021 Apr 20;13:17. doi: 10.1186/s11689-021-09366-1 (PMC8059162; doi:10.1186/s11689-021-09366-1)

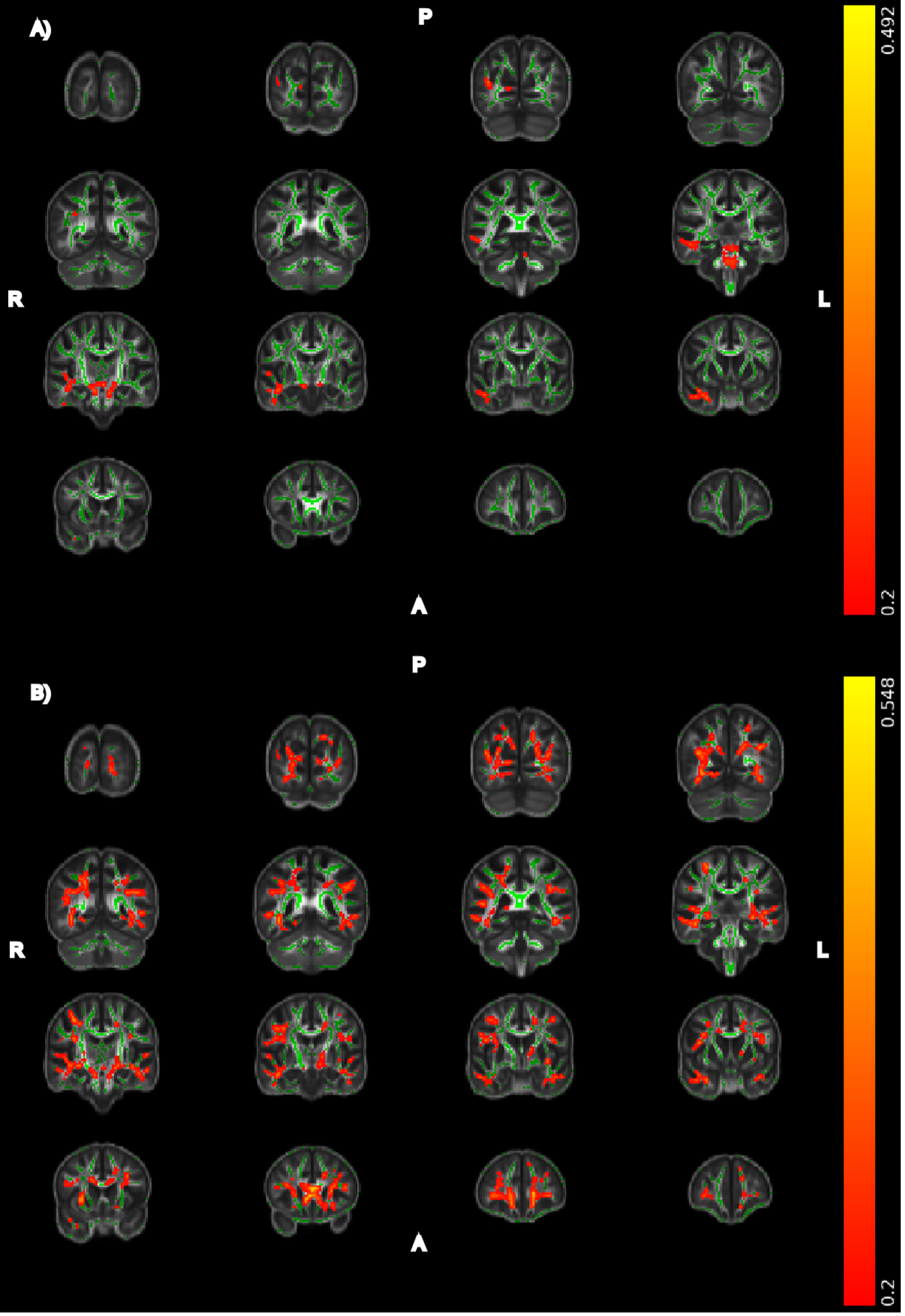

Supplement: Supplementary file 1 — Additional file 1: Supplementary Figure 1. A) Regions of significant positive correlation between EMCS and FA at p < 0.08 FWER corrected and controlling for chronological age, imaging site, and premorbid cognitive ability. B) Regions of significant positive correlation between EMCS and FA at p < 0.05 FWER corrected and controlling for imaging site and premorbid cognitive ability. Images arranged in right to left (R-L) and posterior to anterior (P-A). Images overlaid on the population derived FA skeleton (green) and the population derived 2mm FA template. [file 11689_2021_9366_MOESM1_ESM.png]
